# Experience of unpleasant sensations in the mouth after injection of saline from prefilled syringes

**DOI:** 10.1186/1472-6955-9-1

**Published:** 2010-01-07

**Authors:** Ulf E Kongsgaard, Anders Andersen, Marina Øien, Inger-Ann Y Oswald, Laila I Bruun

**Affiliations:** 1 Department of Anaesthesiology and Intensive Care Medicine, The Norwegian Radium Hospital, Rikshospitalet, Montebello, 0310 Oslo, Norway; 2 The Faculty of Medicine, University of Oslo, 0313 Oslo, Norway; 3 Department of Clinical Pharmacology, The Norwegian Radium Hospital, Rikshospitalet, Montebello, 0310 Oslo, Norway; 4 Department of Anaesthesiology and Intensive Care Medicine, The Norwegian Radium Hospital, Rikshospitalet, Montebello, 0310 Oslo, Norway

## Abstract

**Background:**

Nurses at The Norwegian Radium Hospital have reported that some patients notice an unpleasant smell or taste in accordance with flushing of intravenous lines with commercially available prefilled syringes. We have conducted a study in healthy volunteers to investigate the occurrence, consistency and intensity of this phenomenon.

**Methods:**

A randomised, blinded, crossover study comparing commercial available prefilled saline 9 mg/ml syringes to saline 9 mg/ml for injection in polyethylene package was performed in 10 healthy volunteers. The volunteers were given intravenous injections of varying volume and speed. Data were analysed using descriptive statistics, and also Wilcoxon Signed Rank Test to compare groups.

**Results:**

After intravenous injection, 2 of 15 recordings demonstrated any sensation of smell or taste after injection of saline from polyethylene package, while 14 of 15 recordings noted a sensation after injection of saline from prefilled syringes. The intensity of the unpleasant sensation was rated significantly higher after injection of saline from prefilled syringes compared to saline from polyethylene (p = 0.001).

**Conclusions:**

Injection of saline from prefilled syringes in healthy volunteers resulted in an experience of bad taste or smell. It is important that nurses and health workers are aware of the phenomenon as described in this article in order to choose the preferred product for a given patient.

## Background

In order to maintain the patency of vascular access devices like intravenous cannulaes and central venous lines, these devices are regularly flushed with saline [1]. Commercially available prefilled syringes are in some institutions used to ease the work of the nursing staff. In addition, there have been arguments in favour of these prefilled saline syringes regarding decreased risk of infection and medication errors [2]. In addition, there are organizations and regulatory agencies in some countries that recommend the use of single dose containers for all medications including catheter flushing and locking solutions. However, nurses report that some patients notice an unpleasant smell or taste in accordance with the flushing procedure. This is also mentioned in the Instruction for Use enclosed in the package, where it is noted "Some patients may experience a transitory taste or odour during flushing. This minor effect ceases shortly after the procedure."

We have conducted a study in healthy volunteers to investigate the occurrence of this phenomenon and the consistency and intensity of the experience.

## Methods

A literature search regarding prefilled saline syringes and experience of unpleasant taste, flavour, or odour was carried out. Becton Dickinson Pharmaceutical Systems was contacted for possible background information.

A High performance liquid chromatography (HPLC) analysis of saline from BD Saline XS (Becton Dickinson, 10 ml pre-filled syringe) and from Mini-Plasco^® ^connect (B. Braun, 20 ml polyethylene bottle) was conducted. Fifty microliters of sample was injected on an Agilent 1100 system and separated through a 0.46 × 10 cm C18 Prodigy ODS column using a linear gradient from 10% to 90% acetonitrile in water over 30 minutes. Drift and background was corrected using 18.2 MΩ/cm Milli-Q water).

Then, a randomised, blinded, crossover study comparing prefilled syringes (BD Saline XS, Becton Dickinson [BDs]), to normal saline (from B. Braun, in polyethylene packaging [rSaline]) was performed. 10 healthy volunteers participated, 2 male and 9 female, age 27 to 58. Written informed consent was obtained from all participants. Randomisation list was constructed from Research Randomizer http://www.randomizer.org/form.htm. The volunteers were asked to describe taste and smell after an injection of the two different types of saline (BDs and rSaline). Since this was done in a cross-over fashion (5 volunteers were given BDs - rSaline - BDs, and 5 volunteers rSaline - BDs - rSaline), 15 registrations were recorded for each test. For each experiment we registered taste or smell with Yes or No. We also used a Numeric Rating Scale where 0 represents no bad taste or smell what so ever and 10 represents the worst taste or smell conceivable. The volunteers were given the following categories of smell and taste to describe their experience: Bitter, sweet, sour, salt, metallic, stale, nauseous. The volunteers were also given the opportunity to describe taste/smell with their own words.

The experiment was conducted in the following manner:

An intravenous cannula was inserted into the arms of the volunteers. The volunteers were blindfolded. They were given a 10 ml intravenous injection of BDs or rSaline, in the course of 5 seconds. The volunteers gave feedback on the extent of the smell and/or taste they experienced after receiving the injection. The same procedure for the second and third injection was used. All three injections (of BDs and rSaline) were given in a random order.

After 15 minutes one injection of 10 ml BDs was given to all volunteers within a 30 second period. Finally, (after 15 minutes) one injection of 3 ml of BDs was given to all volunteers within a 3 second period. The purpose with the different injections was to register if the degree of any smell/taste was influenced by low/high injection speed or low/high volume (ml).

The volunteers flushed their mouths with tap water after each test.

The healthy volunteers received 30 € to participate in the project.

The Regional Committees for Medical Research Ethics in Norway and the Norwegian Social Science Data Services approved the study.

### Statistics

SPSS software, version 16.0 (SPSS, Chicago, IL), was used for statistical analyses. Descriptive data and Wilcoxon Signed Rank Test (2-tailed) were applied since normal distribution was not guaranteed and we tested a small number of volunteers. A *P *value < 0.05 was regarded as statistically significant.

## Results

A literature search in PubMed, EMBASE and SveMed using the search terms, Becton Dickinson, prefilled syringes, saline, odour, and taste (single terms and in combination), revealed no published articles addressing this phenomenon.

**Figure 1:**
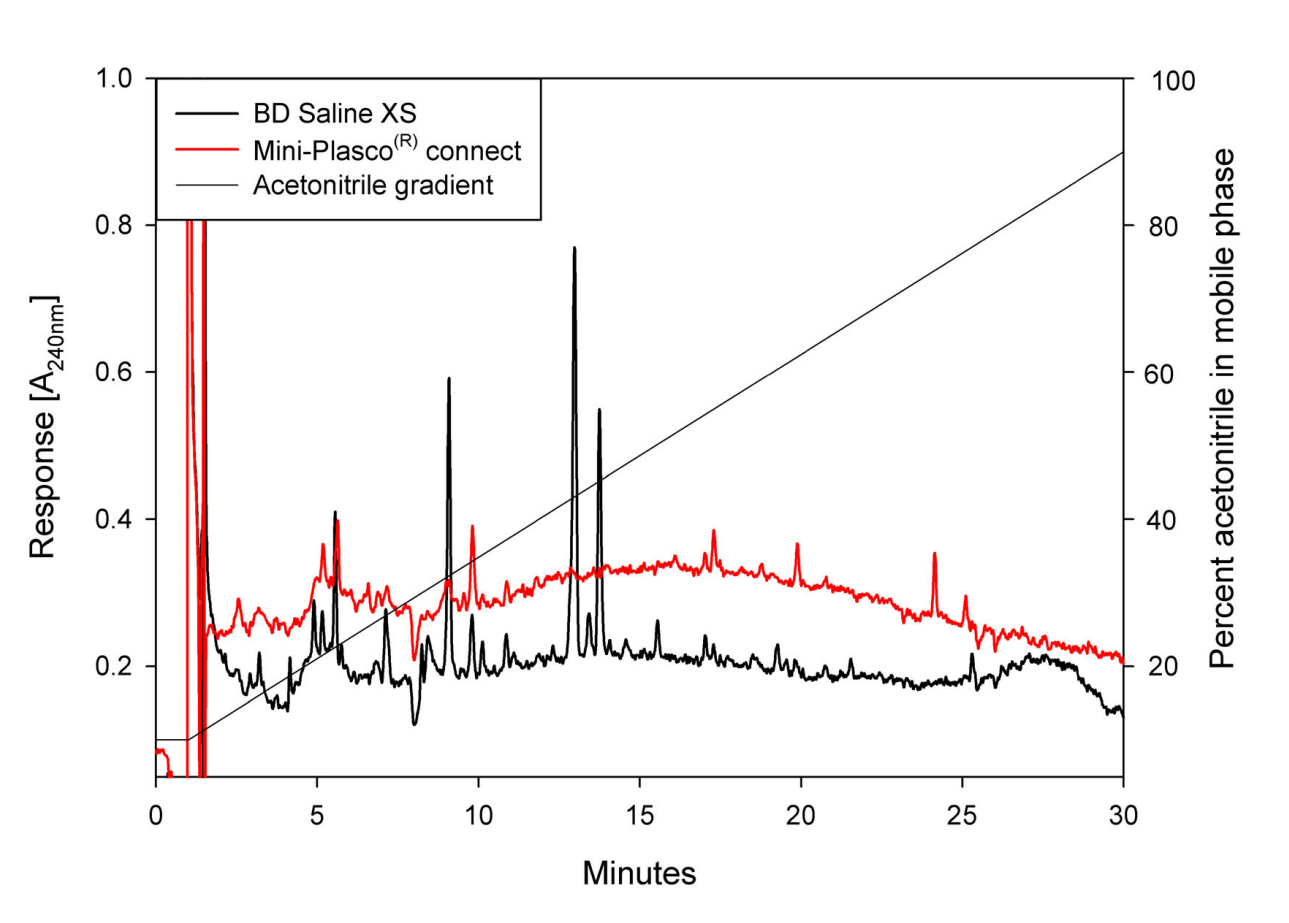
**Chromatograms of saline from BD Saline XS (-) and from Mini-Plasco^® ^connect (-)**. Detection by UV-absorbance at 240 nm. Acetonitrile gradient from 10% to 90% as indicated by solid line. Drift and background was corrected using 18.2 MΩ/cm Milli-Q water.

Chromatograms from HPLC-analysis conducted in our laboratory revealed that both products tested contained a number of unidentified substances generating chromatographic peaks (fig. 1).

Information from Becton Dickinson showed that the presence of volatile substances in the plastic material of the syringes was discovered in 2001 and that these substances were linked to the experience of minor reactions like bad taste or smell. Becton Dickinson concluded that these substances represented no toxic or pharmacological risk to patients' health. The identification and saline-solution concentrations provided by Becton Dickinson were: 2-methyl-2-propanol: 8.5 ppm; 2-methyl-2-butanol: 0.7 ppm; ethyl-buthyl-ether: 0.4 ppm.

**Figure 2:**
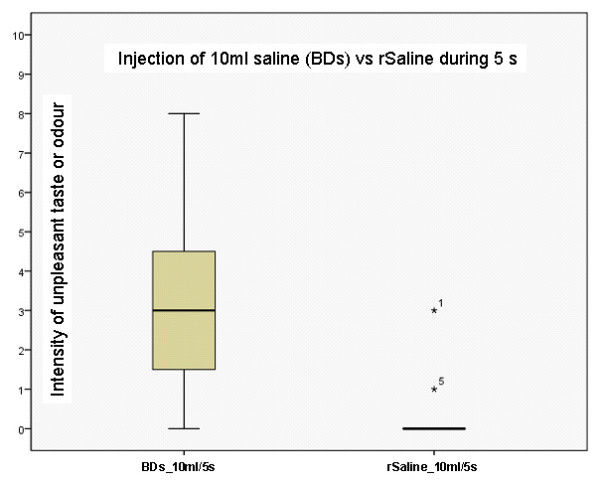
**Box plot of volunteers' experience of intensity of unpleasant taste or odour after injection of 10 ml saline (during 5 seconds) from prefilled syringes from Becton Dickinson (BDs) and regular saline from polyethylene bottles (rSaline)**. Y-axis: Numeric rating scale where 0 = no smell or taste and 10 = worst taste or smell conceivable.

**Figure 3:**
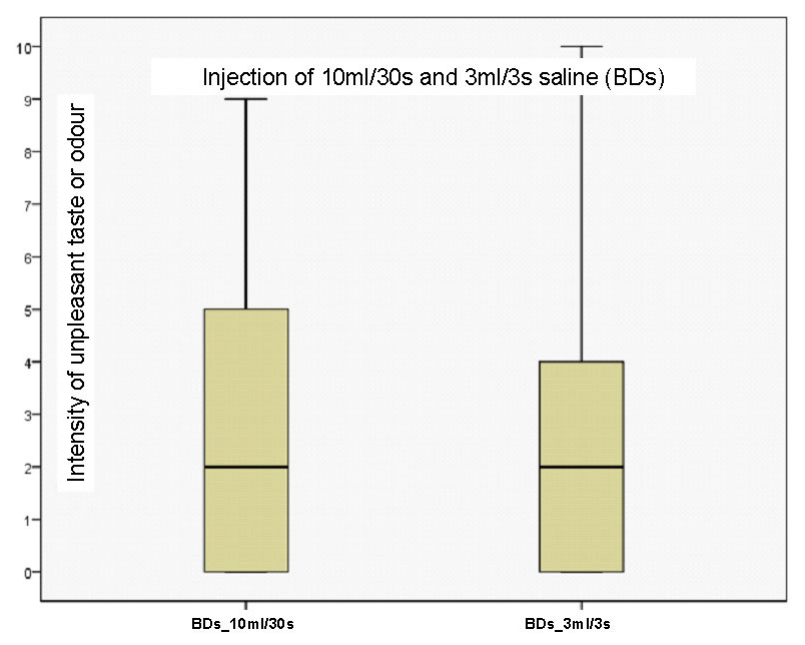
**Box plot of volunteers' experience of intensity of unpleasant taste or odour after injection of 10 ml saline (during 30 seconds) and injection of 3 ml (during 3 seconds) from prefilled syringes from Becton Dickinson (BDs)**. Y-axis: Numeric rating scale where 0 = no smell or taste and 10 = worst taste or smell conceivable.

Two of 15 recordings demonstrated any sensation (smell/taste) after injection of rSaline, while 14 of 15 recordings noted a sensation after injection of BDs. Stale, metallic and sweet were the standard descriptors most used. In addition 4 persons used the word plastic. The intensity of an unpleasant sensation after injection of BDs 10 ml over 5 seconds was rated significantly higher compared to rSaline, 10 ml over 5 seconds (p = 0.001), see figure 2. BDs 10 ml over 30 seconds and BDs 3 ml over 3 seconds are demonstrated in figure 3. Although the sensation when injected at slower speed, or less volume was lower than with 10 ml over 5 seconds, these differences were not statistical significant compared to an injection of 10 ml BDs over 5 seconds.

## Discussion

Injection of saline from BD-syringes resulted in a sensation of bad taste or odour in most volunteers.

The phenomenon of bad taste or smell is explained by the mechanism of volatile substances, released from saline when injected into the bloodstream and then eliminated by the respiratory system and thus detected by the patients in the air expired by the olfactory system. A sensation of taste, mediated by the taste buds, is usually combined with stimulation of touch and smell. Thus it is not surprising that the volunteers confused taste, flavour, and odour.

We cannot find any documentation regarding the frequency or the intensity of this unpleasant experience after flushing of saline with these prefilled syringes. The phenomenon is probably underreported as few patients will find it logical to associate an unpleasant odour with flushing of intravascular devices.

The chromatographic peaks demonstrated in the HPLC-analysis (fig. 1) identify substances in the saline (from BD-syringes) that are not found in regular saline. Whether these peaks represent the volatile substances are likely, but not established.

According to Becton Dickinson, these volatiles substances (2-methyl-2-propanol: 8.5 ppm, 2-methyl-2-butanol: 0.7 ppm, ethyl-buthyl-ether: 0.4 ppm) present no toxic or pharmacological risk to the patient's health even when administered in dosages 1000 times that observed with normal flushing.

However, these effects can be unpleasant for the patients, and might be even worse in nauseated patients whether from anaesthesia or chemotherapy. Probably, the experience is especially disturbing for cancer patients under lengthy chemotherapy who often are more sensitive to taste perceptions [3].

It is claimed from Becton Dickinson that the effect usually can be avoided by using a flushing procedure with a slower flow rate or smaller volume (Becton Dickinson - personal communication). This was not the case in our study. A limitation is the small number of experiences with low injection rate and small volume.

The benefit of prefilled syringes could improve clinician work flow and reduced time required to prepare flush syringes. Furthermore, labeling as part of the manufacturing process increases patient safety. Potential reduction in microbial contamination when used for vascular access devices is also an attractive argument, although documentation from good clinical controlled studies are lacking. However, infectious outbreaks have been associated with improper use of multiple dose vials of saline and heparin. As mentioned previously, different organizations and regulatory agencies in some countries (particularly in the United States) recommend the use of single dose containers for medications including flushing solutions. This means that prefilled syringes are the only options in many places. When weighing the overall risk versus benefits of prefilled syringes, the issue of altered taste could be argued to represent a minor complaint compared to the potential benefits or reduced risk of infection and medication errors.

## Conclusion

Injection of BD-saline in healthy volunteers resulted in an experience of bad taste or smell. When considering the pros and cons of prefilled saline syringes, this phenomenon should be taken into consideration. The manufacturers of these prefilled saline syringes should find ways to reduce the phenomenon.

## Competing interests

The authors declare that they have no competing interests.

## Authors' contributions

UEK participated in all aspects of the project and secured funding. AA helped design the study, performed the HPLC analysis, commented on the results and revised the manuscript. MØ participated in the practical implementation of the project, acquisition of data and commented on the results. I-AYO helped design the study and participated in the practical implementation of the project, acquisition of data and helped draft the manuscript. LIB initiated the project, helped design the study, and commented on and interpreted the results. All authors read and approved the final manuscript.

## Pre-publication history

The pre-publication history for this paper can be accessed here:

http://www.biomedcentral.com/1472-6955/9/1/prepub
